# Subjective and objective refractions in eyes with extended‐depth‐of‐focus intraocular lenses using echelette optics: clinical and experimental study

**DOI:** 10.1111/aos.14660

**Published:** 2020-11-16

**Authors:** Yuka Ota, Keiichiro Minami, Shinichi Oki, Hiroko Bissen‐Miyajima, Keiichiro Okamoto, Masaya Nakashima, Kazuo Tsubota

**Affiliations:** ^1^ Department of Ophthalmology Tokyo Dental Collage Suidobashi Hospital Tokyo Japan; ^2^ Department of Ophthalmology Keio University School of Medicine Tokyo Japan; ^3^ Tomey Corporation Nagoya Japan

**Keywords:** chromatic focal difference, echelette optics, extended‐depth‐of‐focus intraocular lens, near infrared, objective refraction

## Abstract

**Purpose:**

To evaluate differences in subjective and objective refractions in eyes with extended‐depth‐of‐focus intraocular lenses (EDOF IOLs) using echelette optics, and the effect of the light wavelength used during examinations.

**Methods:**

In the prospective study, subjective and objective refractions of 128 eyes of 64 patients were examined 3 months after implantation of the EDOF IOLs (ZXR00V, Johnson & Johnson Surgival Vision). Objective refractions were measured using an autorefractor with a near‐infrared (NIR) light source. Clinical differences in the spherical, cylindrical and spherical equivalent (SE) refractions between the subjective and objective refractions were evaluated. Then, lens powers of monofocal, EDOF and diffractive bifocal IOLs in the use of a 850‐nm light source were measured experimentally for using a lensmeter, and the differences from the monofocal IOLs were calculated.

**Results:**

The mean objective refractions were more myopic (p < 0.001) than the subjective refractions; the differences in the spherical, cylindrical and SE refractions were −0.71, −0.26 and −0.84 dioptre, respectively. Experimental investigation resulted that there was the mean difference of 0.83 D with the EDOF from monofocal IOLs at 850 nm, while the difference was −0.20 D with bifocal IOLs.

**Conclusions:**

The diffractive EDOF IOLs using echelette gratings inherently induced constant differences in the subjective and objective refractions, which arose from the chromatic difference in IOL powers for the visible and NIR lights.

## Introduction

Measurement of the subjective refractions after implantation of presbyopia‐correction intraocular lenses (IOLs) is critical for determining if the postoperative refractions are within the range anticipated preoperatively. Objective refractions measured with an autorefractor are normally used as the initial estimates of the subjective refractions (Kinge et al. 1996; Bullimore et al. 1998). Although these refractions coincide well for eyes with monofocal IOLs, significant differences between the refractive values were found with some refractive multifocal IOLs (Muñoz et al., 2007a, 2007b; Albarrán‐Diego et al. 2011; van der Linden et al. 2014). The lights used during the measurements are disruptive by adding refractions to the IOLs. In contrast, no such refractive differences are observed with diffractive bifocal IOLs (Bissen‐Miyajima et al. 2010), since the diffractive IOLs form the distance focus with the refractive surface of the IOL, which is the same as monofocal IOLs. Thus, the subjective refractions can be measured in the same manner as with monofocal IOLs.

A new concept of presbyopia‐correction IOLs was developed by extending the depth of focus. The Symfony^®^ IOL (Johnson & Johnson Surgical Vision, Santa Ana, CA, USA) is a diffractive extended‐depth‐of‐focus (EDOF) IOL that uses echelette gratings and produces the distance and near foci with the first‐ and second‐order diffractions, respectively (Weeber et al. 2015; Millán & Vega 2017). With EDOF IOLs, a discrepancy between the subjective and objective refractions has been noticed in clinical practices as reported by the manufacturer. The effect of the grating on the objective refraction measurement is concerning. Visible and near‐infrared (NIR) lights are used in measurements of the subjective and objective refractions, respectively, and the power for the distance focus changes with the wavelengths of light used for each examination. To our knowledge, no study has evaluated the difference between the subjective and objective refractions in eyes with diffractive EDOF IOLs. The purpose of the current study was to clinically evaluate the refractive differences and experimentally determine the etiology.

## Methods

Clinical and experimental evaluations were performed in this study. First, the differences were assessed between the subjective refractions obtained from visual acuity examinations and objective refractions measured with an autorefractor after implantation of diffractive EDOF IOLs. In the use of diffractive bifocal IOL in which the distance focus is formed with the refractive element, while the EDOF IOL formed it with the grating element. Hence, the effect of chromatic difference in IOL power for a NIR light on the objective refractions was experimentally examined.

### Difference between subjective and objective refractions

The institutional review board of Tokyo Dental College (identifier, 800) approved this clinical prospective study, which was conducted according to the tenets of the Declaration of Helsinki. Patients who were scheduled to undergo cataract surgery with implantation of a diffractive EDOF IOL (Symfony^®^ ZXR00V) were recruited, and all patients provided written informed consent. The inclusion criteria were anticipated postoperative astigmatism of 1.25 dioptres (D) or less and the conventional criteria for multifocal IOL implantation. Eyes that had undergone a previous ocular surgery, or had chronic or recurrent uveitis, acute ocular disease, external/internal infection, diabetes with retinal changes, glaucoma, exfoliation syndrome, pathological miosis, keratoconus, corneal endothelial dystrophy, or abnormality in the capsule, zonule, or pupil, were excluded. One hundred twenty‐eight eyes of 64 patients were enrolled. Table 1 shows the patient demographic data. The target refractions were emmetropia for 65 eyes and −0.5 D in 63 eyes.

**Table 1:** Patient demographic data.

	Mean (SD)	Range
No. of eyes/patients	128/64	
Age (years)	67.6 ± 6.5	51–82
Axial length (mm)	24.7 ± 1.6	21.6–29.6
Averaged keratometry (D)	43.6 ± 1.1	40.9–46.7
Keratometric astigmatism (D)	0.68 ± 0.38	0.05–1.72

D = dioptre, SD = standard deviation.

#### Implanted intraocular lenses

The ZXR00V IOL was a one‐piece, hydrophobic acrylic, diffractive EDOF IOL that is 6.0 mm in diameter, with aspheric optics on the front surface, a continuous sharp optic edge on the posterior surface, and anteriorly shifted haptic designs. The EDOF function was provided by using the echelette grating on the posterior surface. The add power for the near focus was +1.75 D. In the echelette gratings, the line spacing was larger and the efficacy for higher order diffractions was more optimized compared with the diffractive optics used in bifocal IOLs. Thus, unlike forming a near focus with the first‐order diffraction in bifocal IOLs, the EDOF IOL was designed to form the foci for the distance and slightly near vision with the first‐ and second‐order diffractions. The blazed structure increased the efficacy of the first‐ and second‐order diffractions (Millán & Vega 2017).

The IOLs were implanted during a femtosecond laser‐assisted cataract surgery using the LenSx laser system (Alcon Laboratories, Ft. Worth, TX, USA). The size of capsulotomy was 5.0 mm. The IOL was inserted into the capsular bag using the injector through a 2.2‐mm‐wide corneal incision. With biometry measurement using an IOLMaster 700 (Carl Zeiss Meditec, Inc., Dublin, CA, USA), the IOL powers were calculated using the SRK/T formula (A constant: 119.3) and the Barrett Universal II ( LF: 2.04), and the higher power was selected for avoiding an hyperopic refractive error. The target refraction was emmetropia or −0.5 D in the staged implantation procedure as described previously (Bissen‐Miyajima et al. 2019).

#### Examinations

The subjective refractions were obtained 3 months postoperatively during measurement of the corrected distance visual acuity (CDVA) at 5 m. Without the use of the objective refraction results, the CDVA was measured by increasing the spherical powers in 0.25‐D increments until the corrected visual acuity decreased from the best‐corrected measurement, and the power before the decrease was recorded as the subject’s spherical refraction. The cylindrical power and axis were examined using the cross‐cylinder technique in increments of 0.25 D. The uncorrected distance visual acuity (UDVA) also was measured. The UDVA and CDVA were converted to the logarithm of the minimum angle of resolution (logMAR) for analysis.

Objective refractions were measured with the TONOREF II autorefractor (Nidek, Gamagori, Japan). The instrument utilized the Scheiner principle and projected an image of a circle on the retina, which was observed through the cornea that had been disrupted by the ocular refraction. The light source used was a superluminescent diode emitting in a wavelength in the range of 870–900 nm. The refractive measurement areas were expanded within the entire pupil up to 4.0 mm in diameter. An autofogging mechanism was used to reduce the accommodative effect. The observed ring images were fit with ellipses, and the spherocylindrical refractions were calculated. The spherical equivalent (SE) refraction also was calculated for analysis.

#### Statistical analysis

The differences and correlations in the sphere, cylinder and SE values were examined between the subjective and objective refraction examinations using a paired t‐test and linear regression analysis, respectively. Systematic errors between the subjective (at 5 m) and objective (at infinity) refractions, which corresponded to 0.2 D, were not corrected in the same manner as the previous works (Bissen‐Miyajima et al. 2010; van der Linden et al. 2014). Differences between the two refraction examinations were systematically examined using the Bland–Altman plots (Bland & Altman 1999) for the three refractive values. Data are expressed as the mean ± standard deviation, otherwise specified. p < 0.05 was considered significant.

### Effect of chromatic difference in intraocular lens power

The diffractive EDOF IOL had an echelette optical element, and the first‐order diffraction constituted the distance focus. The 1.75‐D add power was produced in visible light, while the light used in the autorefractor normally has been NIR to retain the intraocular permeability. The diffraction gratings induce negative chromatic aberration, which was used to compensate chromatic aberration induced by monofocal refractive IOL (Weeber et al. 2015). The negative chromatic aberration also increased the refractive powers of the IOL with the wavelength (Ravikumar et al. 2014), so that the objective refraction measurement with NIR light could differ from the amount measured in visible light. The differences were investigated experimentally.

#### Power measurements at 850 nm

Monofocal, bifocal and EDOF one‐piece hydrophobic acrylic IOLs (ZCB00V, ZMB00 and ZXR00V, respectively; Johnson & Johnson Surgical Vision) were used because they have the same lens and haptics platform, except for the optical element for presbyopia correction. IOLs with powers of 10.0, 15.0 and 20.0 D were used for measurements at a wavelength of 850 nm (NIR). Three lenses of each power were examined. A lensmeter (TL‐7000, Tomey, Nagoya, Japan) based on the Hartmann sensor (Hartmann 1900) was modified to accommodate a NIR LED (LSB855SL‐550G; OPTRANS, Latham, NY, USA), which had a peak wavelength of 850 nm, and a CMOS camera (da1280‐54uc; Basler AG, Ahrensburg, Germany).

shows a schematic diagram of the power measurement. Collimated light from the LED illuminated the IOLs that were immersed in water. The focused light passed through the Hartmann plate with 117 pinholes and condenser lenses to the CMOS camera. The CMOS camera captured the pinhole images.

**Fig. 1:**
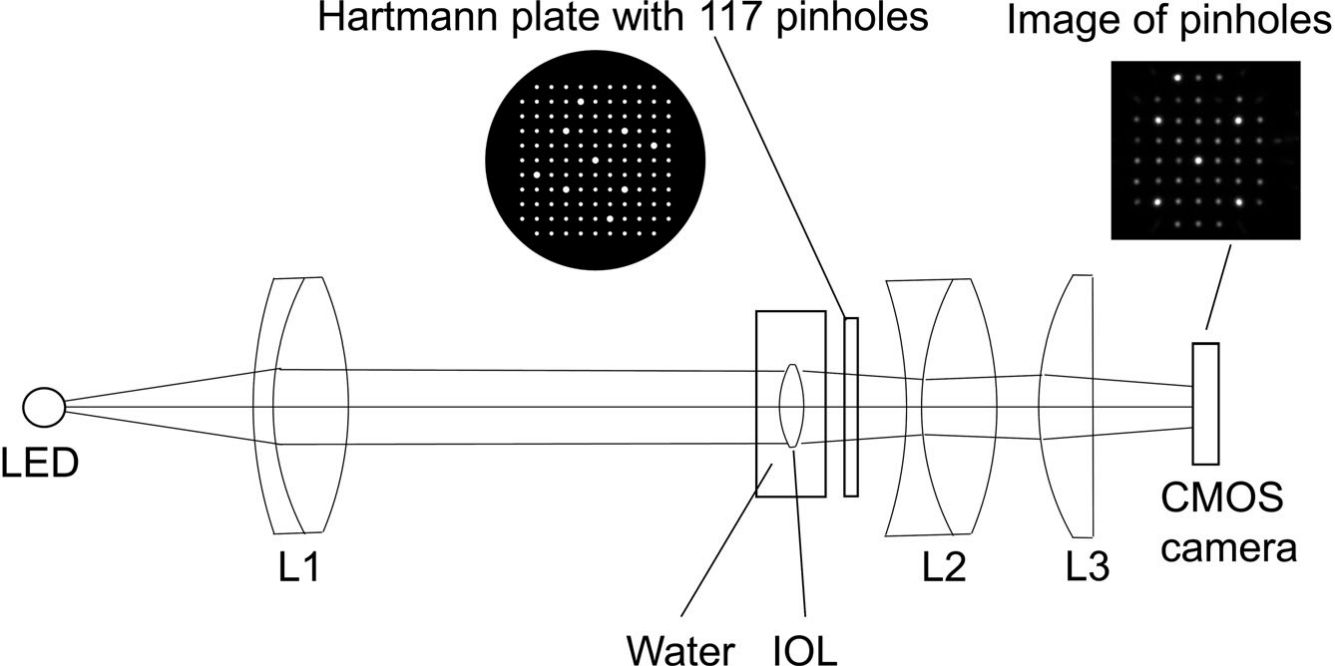
Schematic diagram of the intraocular lens (IOL) power measurement. Lights from a near‐infrared LED (light emitting diode) illuminate the IOL in water after collimated with collimated lenses (L1). Focused lights pass through the Hartmann plate with 117 pinholes and condenser lenses (L2, L3). The pinhole images are captured by the CMOS camera.

**Fig. 2:**
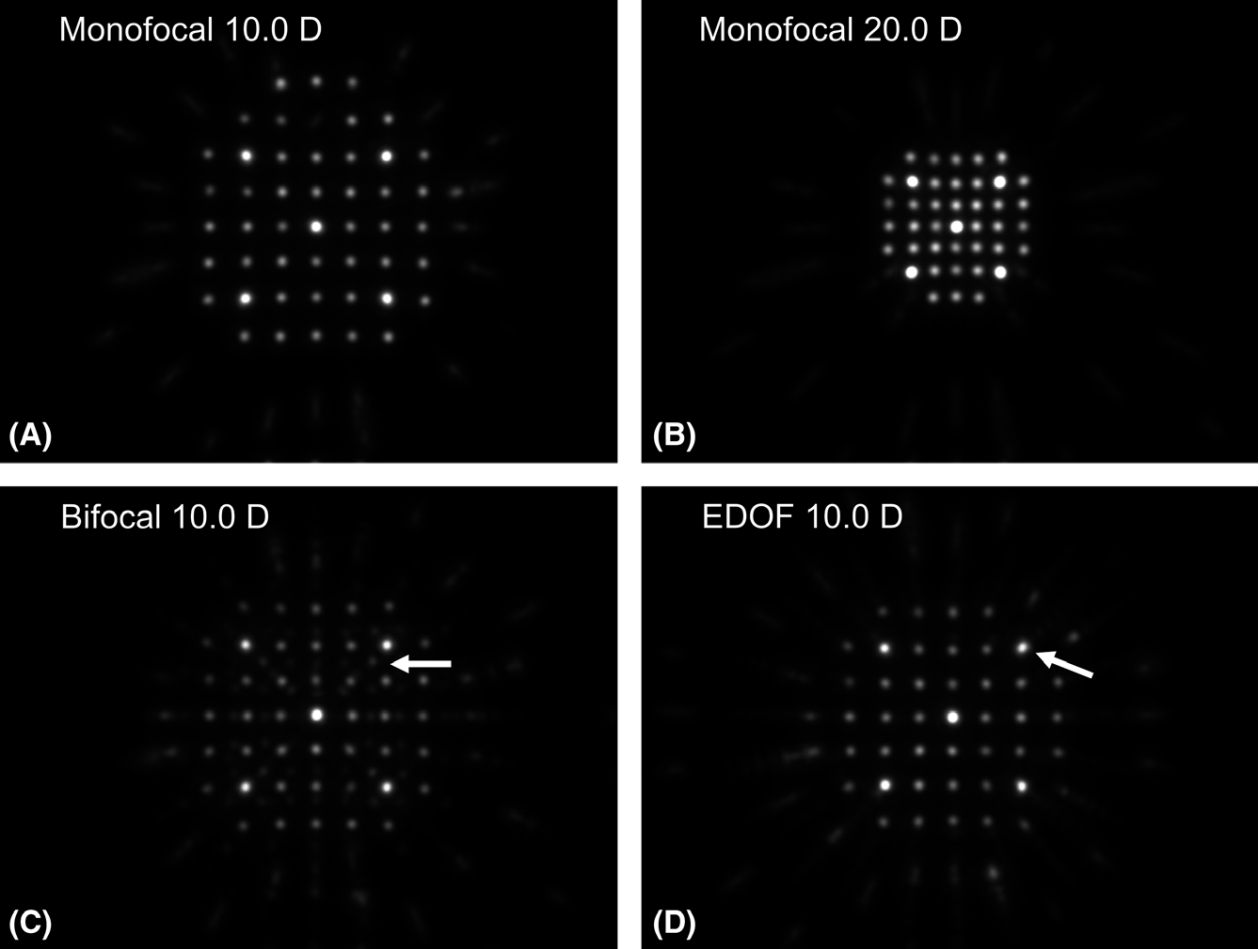
Representative images of Hartmann pinholes with monofocal intraocular lenses (IOLs) of 10.0 D (A) and 20.0 D (B), bifocal IOL of 10.0 D (C), and extended‐depth‐of‐focus (EDOF) IOL of 10.0 D (D) at a wavelength of 850 nm. The distance between the pinholes decreased as the IOL power increased (A and B). A split in the pinhole images (arrow in C) is observed with the bifocal IOL. Slight ovalization (arrow in D) is seen with the EDOF IOL. D, dioptre.

The distance between the pinholes decreased as the power of the IOL increased, as the Hartmann pinholes were posterior to the IOL. Figure 2A,B shows representative images of the pinhole images for the 10.0‐D and 15.0D monofocal IOLs, respectively. Based on geometric optics, the lens powers (D) were negatively proportional to the distance of the pinholes. Six distances between three points around the centre were measured using the ImageJ software (version 1.52p; National Institutes of Health, Bethesda, MD, USA) and averaged. For bifocal IOLs, the points were split towards the centre owing to the +4.0 D add power (Fig. 2C), so that the outer points were subject to measurement. For EDOF IOLs with a +1.75 D add power, the split was too small and the image was slightly oval (Fig. 2D). Therefore, the distances between the outer ellipse foci were measured.

#### Statistical analysis

Differences in the powers of the bifocal and EDOF IOLs from those of the monofocal IOLs were evaluated. First, a calibration equation between the powers labelled and point distances measured was obtained with data with the monofocal IOL using regression analysis. With the calibration equation, the powers of the bifocal and EDOF IOLs at 850 nm were calculated from the pinhole distance data. Differences in the power from the powers of the monofocal IOLs were examined using the Wilcoxon signed‐rank test.

## Results

### Differences between subjective and objective refractions

The postoperative UDVAs ranged from −0.30 to 0.52 logMAR (6/20 to 20/10 in Snellen notation), and the mean was −0.03 ± 0.12. The CDVAs ranged from −0.30 to 0.00 logMAR (20/20 to 20/10 in Snellen notation) with the mean of −0.15 ± 0.05. All eyes obtained a CDVA of 20/20 or better. No intraoperative or postoperative complications occurred. The mean refractions and their differences are shown in Table 2. There were significant differences between the two refractions in the spherical, cylindrical and SE (p < 0.0001, paired *t*‐test). The mean differences showed that the objective refractions shifted myopically, and the SE difference was −0.84 D.

**Table 2:** Mean subjective and objective refractions and their differences.

	Subjective (D)	Objective (D)	p Value*	Difference (D)
Spherical	−0.14 ± 0.42 (−1.00 to 1.00)	−0.85 ± 0.46 (−2.13 to 0.27)	<0.0001	−0.71 ± 0.24 (−1.38 to −0.14)
Cylindrical	−0.45 ± 0.42 (−1.50 to 0.00)	−0.72 ± 0.41 (−1.89 to −0.04)	<0.0001	−0.26 ± 0.22 (−1.29 to 0.15)
SE	−0.37 ± 0.43 (−1.50 to 1.00)	−1.21 ± 0.44 (−2.25 to −0.19)	<0.0001	−0.84 ± 0.24 (−1.51 to −0.32)

D = dioptre, SE = spherical equivalent.

The data are expressed as the mean ± standard deviation (range).

* Paired *t*‐test.

The linear regression analysis between the refractions showed significant (p < 0.001) and strong (*R* ^2^ = 0.74, 0.73, and 0.73) correlations in the spherical, cylindrical and SE refractions, respectively. The coefficients of the resultant regression equations, objective = Slope * subjective + Constant, are shown in Table 3. The slopes were over 0.83, which was close to unity. The constant indicated the differences between the refractions.

**Table 3:** Regression equations of objective spherical, cylindrical and (SE) refractions.

	Slope	Constant (D)
Spherical	0.945 (95% CI, 0.847 to 1.043)	−0.721 (95% CI, −0.765 to −0.678)
Cylindrical	0.837 (95% CI, 0.747 to 0.926)	−0.331 (95% CI, −0.386 to −0.275)
SE	0.868 (95% CI, 0.774 to 961)	−0.891 (95% CI, −0.944 to −0.838)

CI = confidence interval, D = dioptre, SE = spherical equivalent.

**Fig. 3:**
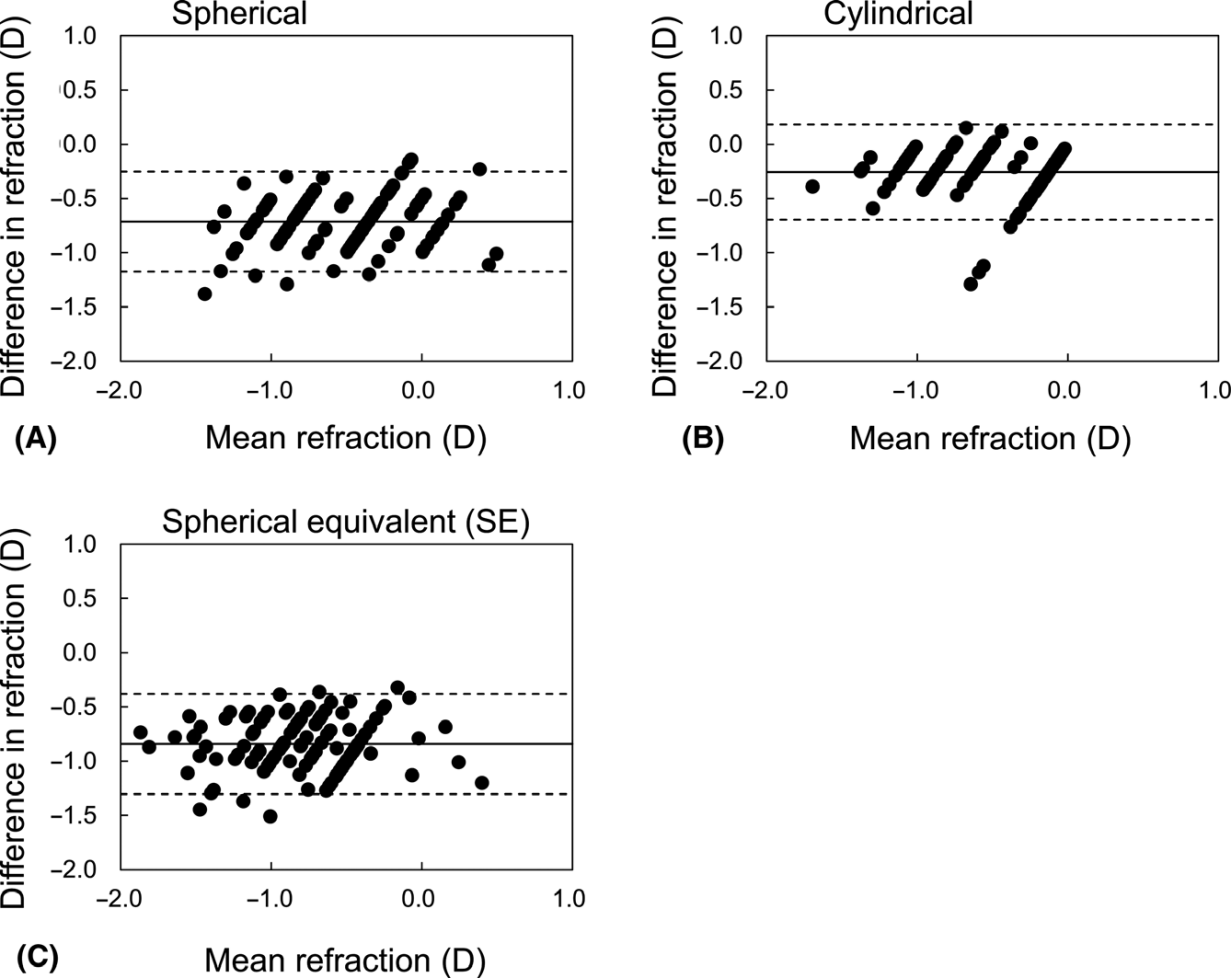
Bland–Altman plots for the spherical (A), cylindrical (B) and SE (C) refractions. The differences in refraction denote the differences between the objective and subjective values. The solid and broken lines indicate the mean and 95% limits of agreement.

Figure 3 shows Bland–Altman plots for the spherical, cylindrical and SE refractions. The *x*‐ and *y*‐axis represented the mean and difference of the subjective and objective refractions, respectively. Systematic differences were found in the spherical and SE refractions. In the cylindrical refraction, the objective refraction agreed clinically with the subjective values.

### Effect of chromatic difference in intraocular lens power

The regression analysis of the data with monofocal IOLs (Table S1 for a list of data with monofocal IOLs) indicated that the distances of the pinholes were significantly and strongly corrected with the power labelled (p < 0.001, *R* ^2^ > 0.999), and the distances decreased by −5.7988 (95% confidence interval [CI], −5.9477 to −5.6499). The powers of the other IOLs were calculated from the distances of pinholes and the above regression result.

Table 4 shows the calculated powers at 850 nm of the bifocal and EDOF IOLs and the differences from the powers of the monofocal IOLs. For the bifocal IOLs, the calculated powers were around the labelled values, and the mean difference from the monofocal IOLs was −0.21 ± 0.19 D. In contrast, the calculated powers were higher than the labelled powers, and the mean difference between the EDOF and monofocal IOLs was 0.83 ± 0.14 D.

**Table 4:** Calculated powers and the differences from the labelled powers of the bifocal and EDOF IOLs.

Labelled power (D)	Wavelength = 850 nm	
	Calculated powers (D)	Differences (D)
Bifocal IOLs		
10.0	10.11, 9.58, 9.97	0.11, −0.42, −0.03
15.0	14.69, 14.61, 14.77	−0.31, −0.39, −0.23
20.0	19.66, 19.98, 19.77	−0.34, −0.02, −0.23
EDOF IOLs		
10.0	10.97, 10.70, 10.70	0.97, 0.70, 0.70
15.0	15.64, 15.78, 15.76	0.64, 0.78, 0.76
20.0	20.97, 21.00, 20.98	0.97, 1.00, 0.98

D = dioptre, EDOF = extended depth of focus, IOL = intraocular lens.

## Discussion

The current evaluations showed that there were differences between the subjective and objective refractions in eyes with diffractive EDOF IOLs. The mean difference was 0.89 D in the SE refractions. The experimental results indicated that the powers of the EDOF IOLs were underestimated when measured at 850 nm, and the difference from the monofocal IOLs was 0.83 D. These findings indicated that the clinical difference in eyes with the EDOF IOLs would result from the chromatic difference in IOL power for NIR lights.

Clinically, the objective refractions for eyes with EDOF IOLs were biased by approximately −1.0 D and the bias did not change. The binocular defocus curve of eyes with the Symfony^®^ EDOF IOLs indicated that the visual acuity ranges from −0.05 to 0.02 logMAR could be obtained between 0.00 D and −1.50 D (Kohnen et al. 2019). To maximize the function of the EDOF IOLs, postoperative refractive errors should be minimized. Ideally, the refractions should be examined without the use of the objective refraction data as in the current study, but the procedure is time consuming. With the current results, the differences inherent in the subjective refraction examination could be compensated for easily.

Since the chromatic aberrations of the diffractive and refractive lenses are opposite each other, diffractive lenses have been used to compensate it inherent in the refractive lens (Davidson et al. 1993; Flores et al. 2004). In the diffractive EDOF IOL, the refractive and diffractive (first‐order diffraction) optical elements were combined for the compensation in visible light (Millán & Vega 2017). The power of the diffractive optical element is determined as 2mλ/*r*_m_^2^ (D), where *r*_m_ indicates the radius of the m^th^ zone in metres and λ is the wavelength of light in metres (Ravikumar et al. 2014). With the calculation, the difference in the EDOF IOL powers at 550 and 870–900 nm was obtained as 1.08–1.11 D, which was close to the experimental result. Thus, the current evaluations showed that the chromatic difference induced by the diffractive element resulted in higher objective refraction measurements using 800‐ to 850‐nm lights.

The current results indicated that the difference between the subjective and objective refractions was inherent from the use of the echelette grating element. For diffractive bifocal IOLs, such a difference has not been found clinically (Bissen‐Miyajima et al. 2010), since the distance focus is formed with the refractive elements. However, in the EDOF IOL, the distance focus was formed with the first‐order diffraction of the echelette grating element, so that the effect of the difference in IOL power would be enhanced at the 800–850 nm wavelength. It was supposed that the effect resulting from the refractive lens would be relatively small because the Abbe number (55) and refractive index (1.47) of the IOLs were relatively low (Millán & Vega 2017). In autorefractors, the difference between the visible and NIR light should be corrected for refractive lenses. However, diffractive lenses have been rarely used for pseudophakic eyes, so that the difference found in the EDOF IOL would not have been addressed and corrected. The use of diffracted light to form the distance focus is also seen in diffractive trifocal IOLs. The PanOptix^®^ IOL (Alcon) is based on a quadrifocal design using three add powers, one of which is designed for the distance focus together with the refractive of the IOL base curve (Sudhir et al. 2019). Therefore, the effect on the objective refraction is concerning. A clinical evaluation of 62 eyes that were implanted with the IOLs and examined with an auto kerato‐refractometer KR‐8800 (Topcon, Japan, NIR light source) showed no difference in the spherical refraction between the subjective and objective refractions (Rementería‐Capelo et al., 2020), whereas the objective cylindrical and SE refractions shifted myopically (mean differences, −0.43 and 0.20 D, respectively). An examination of the differences in the powers at 550 and 800–900 nm is required to investigate the effect.

The current study had some limitations. Clinically, only the autorefractor was used for the objective refraction, while could have been measured using other instruments, such as a Shack–Hartmann aberrometer, automatic retinoscopy, ray tracing and Tscheming technologies (Rozema et al. 2005; Haddad et al. 2020). The wavelengths of lights used in these measurements were red light (650–785 nm) and NIR (808–850 nm), so that the influence of the diffractive optical element would alter. Further examinations are necessary for confirmation. Next, there were systematic errors between the subjective and objective refractions (0.2 D) owing to the 5‐m measurement of subjective refraction. The subjective examinations for infinity are impractical. In a previous evaluation of bifocal IOLs, the mean differences in the sphere and SE refraction were −0.12 and −0.28 D (Bissen‐Miyajima et al. 2010), whereas the subjective refraction was examined in increments of 0.25 D. The effect would be clinically negligible. In the experiment, the areas of the clinical and experimental measurements were not comparable. The experimental measurement was analysed around nine central points, so that the results would represent values on the optical axis. However, the autorefractor measured a wider area of 2–4 mm in diameter. The corneal keratometry would change from the centre to the surroundings, and the difference involves an error in the objective refraction. Corneal topographic data measured using anterior‐segment optical coherence tomography are necessary to compensate.

In conclusion, the diffractive EDOF IOLs using echelette gratings inherently induced constant differences between the subjective and objective refractions, and the differences resulted because the first‐order diffraction formed the distance focus and the powers at visible and NIR lights differed due to the chromatic difference in IOL power.

## Supporting information


**Table S1.** Mean distances of three‐pinhole images with monofocal IOLs.
