# Supplementary material for: Subjective and objective refractions in eyes with extended‐depth‐of‐focus intraocular lenses using echelette optics: clinical and experimental study
Source: Acta Ophthalmol. 2020 Nov 16;99(6):e837–43. doi: 10.1111/aos.14660 (PMC8518701; doi:10.1111/aos.14660)
Supplement: Supplementary file 1 — Table S1. Mean distances of three‐pinhole images with monofocal IOLs. [file AOS-99-e837-s001.docx]

**Table S1**. Mean distances of three-pinhole images with monofocal IOLs

| Labeled power (D) | Distance between 3 pinholes (pixel) |
| --- | --- |
|  | Wavelength = 850 nm |
| 10.0 | 144.3, 143.7, 145.0 |
| 15.0 | 116.9, 115.8, 116.4 |
| 20.0 | 85.8, 86.3, 87.0 |

D, diopter; IOL, intraocular lens.
